# *In-silico* Antigenicity Determination and Clustering of Dengue Virus Serotypes

**DOI:** 10.3389/fgene.2018.00621

**Published:** 2018-12-07

**Authors:** Jingxuan Qiu, Yuxuan Shang, Zhiliang Ji, Tianyi Qiu

**Affiliations:** ^1^School of Medical Instrument and Food Engineering, University of Shanghai for Science and Technology, Shanghai, China; ^2^Shanghai Qibao Dwight High School, Shanghai, China; ^3^State Key Laboratory of Cellular Stress Biology, School of Life Sciences, Xiamen University, Xiamen, China; ^4^Shanghai Public Health Clinical Center & Institutes of Biomedical Sciences, Shanghai Medical School, Fudan University, Shanghai, China

**Keywords:** dengue virus, envelope protein, bioinformatics, antigenicity-dominant positions, antigenicity clustering

## Abstract

Emerging or re-emerging dengue virus (DENV) causes dengue fever epidemics globally. Current DENV serotypes are defined based on genetic clustering, while discrepancies are frequently observed between the genetic clustering and the antigenicity experiments. Rapid antigenicity determination of DENV mutants in high-throughput way is critical for vaccine selection and epidemic prevention during early outbreaks, where accurate prediction methods are seldom reported for DENV. Here, a highly accurate and efficient *in-silico* model was set up for DENV based on possible antigenicity-dominant positions (ADPs) of envelope (E) protein. Independent testing showed a high performance of our model with AUC-value of 0.937 and accuracy of 0.896 through quantitative Linear Regression (LR) model. More importantly, our model can successfully detect those cross-reactions between inter-serotype strains, while current genetic clustering failed. Prediction cluster of 1,143 historical strains showed new DENV clusters, and we proposed DENV2 should be further classified into two subgroups. Thus, the DENV serotyping may be re-considered antigenetically rather than genetically. As the first algorithm tailor-made for DENV antigenicity measurement based on mutated sequences, our model may provide fast-responding opportunity for the antigenicity surveillance on DENV variants and potential vaccine study.

## Introduction

Dengue virus (DENV) is a mosquito-borne RNA virus from flaviviridae family, which could cause dengue fever epidemics in tropical and subtropical countries (Rodenhuis-Zybert et al., 2010). Every year, nearly 390 million people were infected by DENV, among them, 96 million developed into an acute systemic illness and over 500 thousand experienced potentially life-threatening complications such as dengue hemorrhage fever (DHF) and dengue shock syndrome (DSS) (WHO/TDR, 2009; Bhatt et al., 2013). Traditionally, DENV are genetically divided into four subtypes (Lanciotti et al., 1997; Zhang et al., 2005; Chau et al., 2008). In 1952, an early clinical study reported that individuals with primary DENV infections often provide protections among the homologous type, and show only partial cross-protection against heterologous types (Sabin, 1952). As such, DENV serotypes were simply defined based on genetic clusters. This classification was subsequently supported by *in vitro* experiments in which DENV strains were better neutralized by antisera from homologous rather than those of heterologous types (Hammon et al., 1960). Despite above, it was frequently realized that antigenic variation does occur within same DENV serotype. Initially, this intra-serotype difference was considered as substantially less than those of inter-serotypes, and can be neglected (Russell and Nisalak, 1967; Gentry et al., 1982). Yet, with the accumulation of clinical and epidemiological evidence, researchers noted that the traditional classification of DENV serotypes based on genotypes can no more explain the clinical observations. Cross-reactions were often found between antiserum from different serotypes, which leads to the rethinking of the DENV antigenicity clustering (Katzelnick et al., 2015). Currently, it was believed that the antigenicity of DENV viruses was actually volatile, while the traditional genotypic categorization may not be sensitive enough to evaluate the antigenicity difference (Katzelnick et al., 2015). Also, the epidemic magnitude of DENV might not only be affected by traditional serotypes, but most importantly, be determined by antigenic differences between particular infecting viruses (Kochel et al., 2002; Adams et al., 2006; OhAinle et al., 2011). Since antigenic differences among the DENV types correlate with not only disease outcome and vaccine-induced protection, but also epidemic magnitude and viral evolution, accurate antigenic analysis were highly desired for DENV serotypes.

In order to investigate the antigenicity relationship of DENV subtypes, comprehensive serological tests were accomplished on both animal and vaccinated or infected humans to calibrate the serological relationship between DENV subtypes. In Leah's study, 36 DENV isolates covering four serotypes were selected to inoculate against African green monkey, and the anti-serum of each monkey was tested against 47 DENV strains to generate dengue antigenic mapping (Katzelnick et al., 2015). According to antigenic cartography, the antigenicity of DENV isolates are usually similar to those viruses from the same serotypes. However, a substantial number of strains illustrated greater antigenic variance to inter-type viruses than those from intra-types (Katzelnick et al., 2015).

Above results suggest that the traditional genotype classification cannot fully meet the needs of antigenicity clustering, and new methods of more accurate antigenicity evaluation are highly needed. With the development of bioinformatics technology, computational approaches have started to provide possibility in both accurate and high-throughput way (Liao et al., 2009; Qiu et al., 2016). Although, this may be managed by a few general *in-silico* model (Qiu et al., 2018), the limitation often includes requiring clearly defined epitope residues and low computational efficiency. In this study, a rapid model tailor-made for DENV was established to infer the antigenic relationship between inter- and intra-serotypes of DENV strains considering the conformational environment of major surface envelope (E) protein. Based on the comprehensive experimental dataset collated from previous researches, antigenicity-dominant positions (ADPs) of DENV and four serotypes were firstly derived based on the correlation between residual mutation of E protein and antigenicity variance. Then, the position specific scoring matrix (PSSM) was combined with physic-chemical descriptors (PCDs) to build the antigenicity calculation model. Finally, 1,143 historical sequences of DENV E antigens from NCBI (Resch et al., 2009) were predicted and the antigenicity relationship was analyzed between DENV serotypes.

## Materials and Methods

### Dataset

For model construction, virus-antiserum neutralization titers which reflecting the antigenic relationship were collated from previous researches (Katzelnick et al., 2015), in which the binding ability between DENV and DENV-post-infection African green monkey antisera were determined. Corresponding envelope protein of DENV were collected from National Center for Biotechnology Information (NCBI) (Resch et al., 2009). Considering the injected time and integrity of data, antisera samples derived from African green monkey which injected with corresponding vaccine for 3 mouth were chosen for model construction and validation. Totally, 1,444 strain pairs with experimental antigenicity distance involving 46 strains were retained and those with antisera value labeled as <10 were arbitrarily set as 5 to simplify the calculation. For model construction, 80% of strain pairs (1,155) from experimental data were randomly selected as training dataset and the remaining 20% (289 strain pairs) were defined as independent validation set.

Further, historical DENV strains with envelope protein sequence were collected virus variation resources at the NCBI (Resch et al., 2009), a total number of 4,633 E protein sequences were retained. Based on the sequence identity of 100%, 1,143 un-redundant E protein sequence were selected for further analysis. The three-dimensional structure of envelope protein was collated from Protein Data Bank (PDB id: 1OAN) (Berman et al., 2000; Modis et al., 2003).

### Identifying Antigenicity-Dominant Positions of E Protein Surface

Since the antigenicity recognition between antigen and antibody often occurs at the interaction interface of antigen surface, those surface mutations exposed on protein surface in training set were initially selected as candidates. After mapping all positions to template structure (PDB id: 1OAN), 357 surface positions are collected with solvent accessible surface areas (SASA) over 1 Å, which was calculated through Naccess V2.1.1. As antigenic variation often related with mutations at multiple positions, it can be further correlated with antisera titer values by linear regression (LR).

For each strain pair to be compared, the candidate ADPs are defined as set *P*, which initially covers 357 surface positions. By marking the positions with amino acid mutations as 1 and otherwise as 0, a 357-bit vector *vec*(*P*) can be generated. Combined with the normalized antisera titer value, a LR was established and those positions with weight (absolute value) over 0 was defined as positions correlated with antigenicity distance. In that case, 97 ADPs were retained. According to geometric distance, those 97 positions can be classified into four antigenic patches. Here, antisera titer value (*V*) was normalized by logarithm (*Log*_2_*V*). For individual serotypes, the ADPs were derived based on intra-serotypes experimental titers.

### Quantitative Model Construction Based on Antigenicity-Dominant Positions

#### Position Specific Scoring Matrix

To quantitatively describe amino acid mutations on each antigenic dominant position, amino acid distribution was calculated to reflect the effect of residue mutations. A PSSM was generated by position-specific iterated basic local alignment search tool (PSI-BLAST) (Altschul et al., 1997) based on 1,143 historical envelope protein sequence. Each score on a 1 × 20 matrix represents the frequency of each amino acid occurred on the described positions. For a pair of DENV strains, PSSM vector was constructed based on the score of each position, each score was defined as absolute difference of matrix score for compared residues. For each queried E protein pairs, a 97-bit PSSM descriptor was formulated to summarize amino acid mutations at 97 positions.

#### Physical Chemical Property Descriptors

Physical chemical property descriptors were based on amino acid index from AAindex database (Kawashima and Kanehisa, 2000). The optimization of physic-chemical indexes was done as below: (1) Pair-wised Pearson Correlation Coefficient (PCC) were calculated between any two AAindexes. (2) Two indexes were defined as similar only when the corresponding PCC-value was over 0.8. (3) All indexes were ranked according to the number of similar ones which can be represented in descending order. (4) From the top to the bottom of rank list, indexes which can be represented by others were removed sequentially. (5) Minimum index set was obtained which can represent the full index list. Physic-chemical property descriptor was generated based on the absolute difference of AAindex summed for each antigenic region, further, the relationship between PCDs, and experimental titers were constructed through LR for feature selection. Here, the neighborhood region of conformational structures was set as 1 Å according to distance screen from 1 to 5 Å (Supplementary Table 2). Each round, those indexes with weight unequal to 0 were remained and after iterative selection, 20 antigenically-related indexes were selected for further model construction. For the four antigenic patches, (4^*^20 =) 80 bits of descriptors were generated as physic-chemical property descriptors.

#### Modeling the Antigenic Variance

Based on antigenic descriptors incorporating PSSM profile and physic-chemical properties, prediction model for antigenicity regression could be constructed. Here, both qualitative and quantitative model were adopted for model construction between normalized experimental titers and antigenicity descriptors. To further analysis the antigenic relationship, different antigenic cutoffs were set for classifications based on the homologous titers between DENV strains and the antiserum against itself. In that case, for the strain pairs based on E protein marked as *E*_*a*_ and *E*_*b*_, a 177-array quantitative descriptor for antigenicity-dominant positions (QDAP) was derived as below containing PSSM profiles and PCD. Further, the machine learning model can be generated to fit the parameters of 177-dimensional descriptors for antigenic variation which defined by logarithm of experimental titers (*LogV*_*ab*_) on the training set, as follows:

(1) $$\left\{ \begin{array}{l} \;QDAP_{1:177}^{\left(E_{a},E_{b}\right)}=\left\{PSSM_{1:97}^{\left(E_{a},E_{b}\right)}+PCD_{1:80}^{\left(E_{a},E_{b}\right)}\right\}\\ LogV_{ab}=Train_{1155}\left(\alpha_{1}QDAP_{1},\alpha_{2}QDAP_{2}\cdots \alpha_{177}QDAP_{177}\right)+\varepsilon_{\alpha } \end{array}\right.$$

Till the optimized model is reached as below:

(2) $$\hat{LogV_{ab}}=\gamma_{0}+\gamma_{1}QDAP_{1}+\gamma_{2}QDAP_{2}+\cdots +\gamma_{177}QDAP_{177}$$

Here, score $\hat{LogV_{ab}}$ stood for the predicted antigenicity variation between two DENV strains. The experimental *LogV*_*ab*_ represent the logarithm of experimental titers which used for model training. Thus, the escape threshold for the predicted $\hat{LogV_{ab}}$ is the same as that of *LogV*_*ab*_ from experimental titers.

### Parameter Definition

To evaluate the performance of our model, statistical parameters were defined as follows:

(3) $$\text{Accuracy }=\frac{TP+TN}{TP+FP+TN+FN}$$

(4) $$\text{Precision}=\frac{TP}{TP+FP}$$

(5) $$\text{Sensitivity}=\frac{TP}{TP+FN}$$

Here, TP represents true positive, TN represents true negative, FP represents false positive, and the FN represents false negative. Also, to evaluate our regression mode, correlation coefficient (CC) was introduced as follows:

(6) $$Correlation\;coefficient=\frac{\sum_{i=1}^{n}\left(X_{i}-\bar{X}\right)\left(Y_{i}-\bar{Y}\right)}{\sqrt{\sum_{i=1}^{n}{\left(X_{i}-\bar{X}\right)}^{2}}\sqrt{\sum_{i=1}^{n}{\left(Y_{i}-\bar{Y}\right)}^{2}}}$$

Where *X*_*i*_ represents the predicted value, *Y*_*i*_ represents the actual value, $\bar{X}$ refer to average of *X*_*i*_, and $\bar{Y}$ refer to the average of *Y*_*i*_.

## Results

### Determination of Antigenicity-Dominant Positions

**Figure 1:**
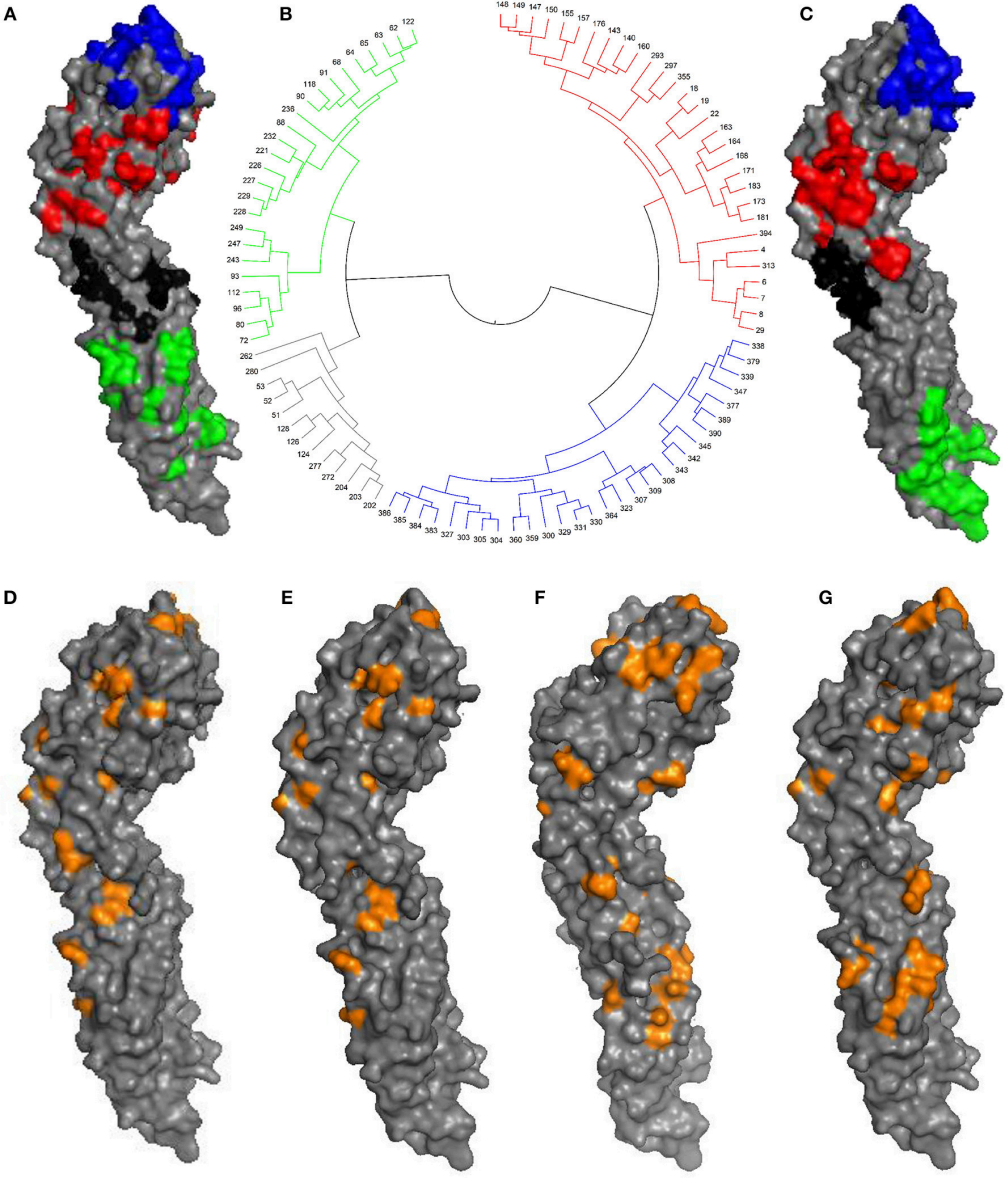
Antigenic-dominant positions of DENV. **(A)** The 97 ADPs mapped on envelope (E) protein (PDB id: 1OAN). D1, D2, D3, and D4 patches were marked in blue, red, black, and green, respectively. **(B)** Clustering tree based on geometric distances between 97 ADPs, antigenic regions were marked with colors. **(C)** Broad-neutralizing epitope areas previously determined for E protein (Aaskov et al., 1989; Cockburn et al., 2012; Fibriansah et al., 2014, 2015). **(D)** 30 ADPs for DENV serotype 1. **(E)** 40 ADPs for DENV serotype 2. **(F)** 47 ADPs for DENV serotype 3. **(G)** 37 ADPs for DENV serotype 4.

Antigenicity-dominant positions (ADPs), whose mutations are correlated with antigenicity variation, were determined by following procedures: (1) surface exposed residues with potential to become epitopes for immune response, and (2) essential positions where mutations will likely lead to antigenicity variations (see section Materials and Methods). Three hundred and fifty-seven surface exposed positions were initially retrieved. According to the correlation with training data from experimental antigenicity variance (Katzelnick et al., 2015), 97 were identified as potential ADPs. It can be found that ADPs are mainly located in domain ED1, ED2, and ED3 on E protein surface, which was illustrated in Figure 1A. Above ADPs can be clustered into four major surface patches according to spatial distance, which may correlate to potential epitope areas on E protein. Here, all four domains were labeled as D1, D2, D3, and D4, as being marked in blue, red, black, and green in Figure 1B, respectively.

It can be seen that those 97 ADPs are highly overlapping with broad-neutralizing epitopes derived from corresponding antibodies targeting all four serotypes (Figure 1C). For instance, the cross-neutralizing mAb of 4E11 was reported to recognize the ED3 region of E protein monomer structure (Cockburn et al., 2012), and another mAb of 1F4 could bind to ED1 regions (Fibriansah et al., 2014). They are well-matched with our region of D1 (blue) and D2 (red), respectively. Also, cross-neutralizing antibodies for polymer structures, such as 1B7 (Aaskov et al., 1989) and 2D22 (Fibriansah et al., 2015), are partially overlapping with our region of D3 (black) and D4 (green).

Besides the general ADPs for DENV, serotype-specific antigenic sites were also determined for each DENV serotype according to antisera of corresponding serotypes in a similar way (see section Materials and Methods). Finally, 30, 40, 47, and 37 sites are derived as serotype-specific ADPs for DENV 1–4, respectively, as been illustrated in Figures 1D–G and Supplementary Table 1.

### Model Construction and Evaluation

Machine learning models are adopted here for DENV antigenicity predictions. Molecular features mainly cover positions specific scoring matrix (PSSM) and physic-chemical environments for each of the ADPs, which were previously reported important to antigenicity predictions (Qiu et al., 2016, 2018). The workflow of DENV antigenicity prediction model covers four steps: (1) deriving PSSM for each ADP, (2) generating physic-chemical properties of neighboring regions for each ADP clusters, (3) selecting appropriate machine learning approaches, and (4) calculating the antigenicity distance between two compared DENV strains. Detailed information can be found in section Materials and Methods.

**Figure 2:**
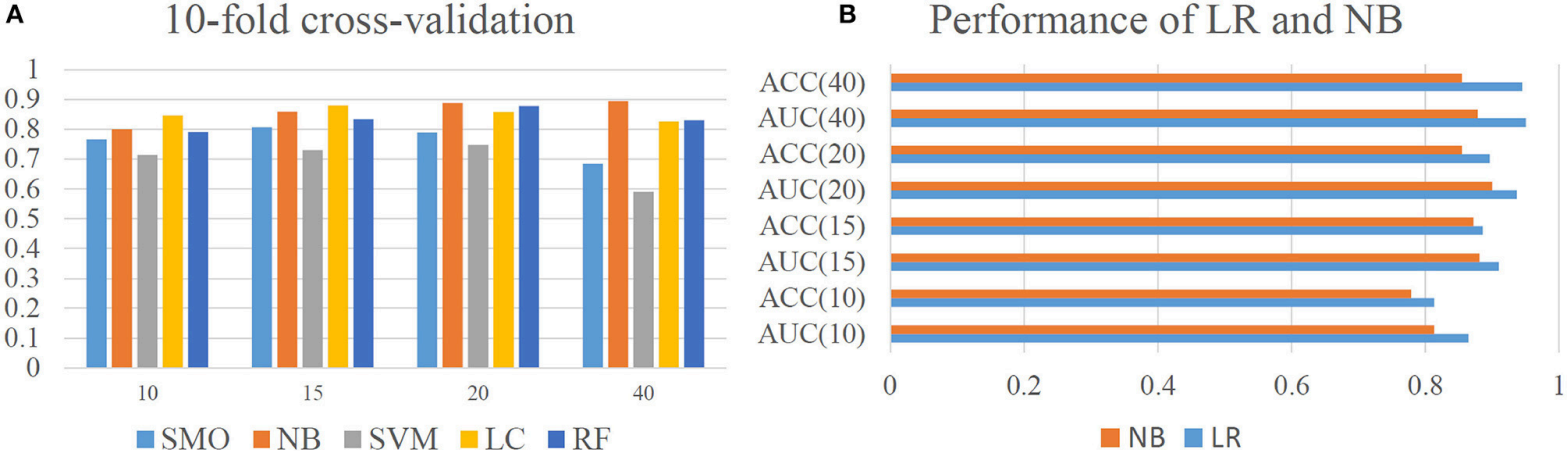
Model performance of our model. **(A)** Cross-validation performance of qualitative model constructed by Sequential Minimal Optimization (SMO), Naïve Bayes (NB), Support Vector Machine (SVM), Logistic Classifier (LC), and Random Forest (RF). Here, Y axis represents the AUC-value of different computational models. **(B)** Performance of Linear Regression and Naïve Bayes on independent dataset.

For machine learning methods, both qualitative and quantitative approaches were tested. Five qualitative models including Sequential Minimal Optimization (SMO), Naïve Bayes (NB), Support Vector Machine (SVM), Logistic Classifier (LC), and Random Forest (RF) were used to establish different classification models. Note that, no titer threshold has been reported in DENV case. According to experimental results of Katzelnick's (Katzelnick et al., 2015), over 90% of self-reactive titer value is over 20. In that case, tilter value of 10, 15, 20, and 40 were tentatively tried in turn as classification cutoff for evaluation. Through 10-fold cross-validation, the performance of all five algorithms indicated that NB classifier obtained the best overall performance on different thresholds and achieved the AUC-value over 0.88 under the threshold of 20 (Figure 2A). Also, the average (AVG) accuracy of our model achieved a range from 0.763 to 0.931 and fluctuation of accuracy is extremely small with variance (VAR) no more than 0.002 (Supplementary Figure 1). This results illustrated that our model could provide an accurate and robust prediction on antigenic classifications and NB classifier was chosen to establish our qualitative mode. After that, the performance of our model was evaluated through independent testing dataset from previous experiments (Katzelnick et al., 2015). Results indicated that our NB classifier could achieve high AUC from 0.81 to 0.90 and ACC from 0.77 to 0.86 under different thresholds, which indicate the outstanding ability of our model for qualitative antigenicity classifications between comparable DENV strains (Supplementary Figure 2).

For quantitative approaches, different regression model including Additive Regression (AR), Support Vector Regression (SVR), Gaussian Processes (GP), LR, and Isotonic Regression (IR) were evaluated. Results indicated that LR could achieve the best quantitative predictions with CC of 0.744 (Supplementary Figure 3). Thus, LR was chosen to establish our quantitative model. Further, by setting different thresholds, the classification performance of quantitative LR model was also evaluated and compared with qualitative NB classifier (Figure 2B). The results showed that quantitative LR model are always better than qualitative NB classifier under different thresholds. Thus, quantitative model of LR was adopted for final analysis.

### The Discrepancy Between DENV Serotypes And Antigenicity Clusters

**Figure 3:**
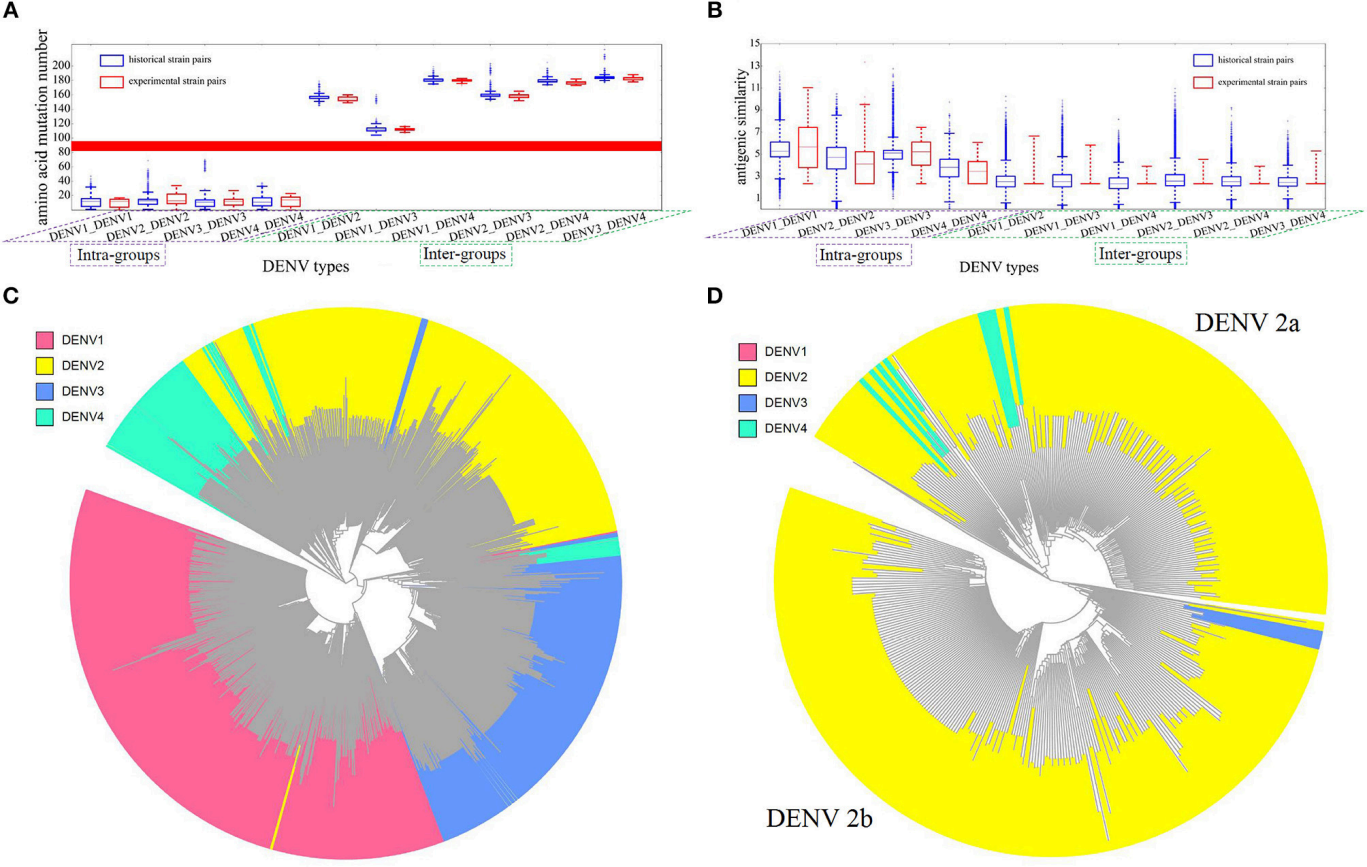
Antigenicity and sequence analysis of DENV. **(A)** Genetic distance between intra- and inter-DENV serotypes by the number of residual mutations. Blue bars represent the results of 1,143 historical strains, red bars represent the experimental strains of 47 strains from Leah's work (Katzelnick et al., 2015). X axis represents the four intra-groups and six inter-groups, Y axis represents mutation abundancy of compared strain pairs. **(B)** Antigenic similarity between intra- and inter- DENV strains. Blue bars represent the results of 1,143 historical strains, red bars represent the experimental binding results of 47 strains from Leah's work (Katzelnick et al., 2015). X axis represents four intra- groups and six inter-groups. Y axis represents antigenic similarity of compared strain pairs, represented by logarithm of titer value. **(C)** Antigenicity clustering of 1,143 DENV strains, red, yellow, blue, and cyan represents DENV serotype 1, 2, 3, and 4, respectively. **(D)** Antigenicity clustering of two major clusters of DENV serotype 2, including DENV 2a and DENV 2b.

With above model, we made a large-scale antigenicity mapping for 1,143 historical DENV strains retrieved from NCBI to investigate the relationship between DENV serotypes (genetic clusters) and antigenicity clusters. Firstly, the pair-wised antigenicity similarity of all 1,143 historical strains were calculated through our model for intra- and inter-serotypes. Similarly, the genetic distance was also done by counting the number of residual mutations for intra- and inter-serotypes (Figure 3). It can be seen that, the genetic distance or variation within one serotype is significantly less than that of inter-serotypes and the distribution ranges of genetic distance were clearly distinguishable without any overlapping between the two classes (Figure 3A). However, in the case of antigenicity similarities, this border become overlapping, despite the slight differential trends (Figure 3B). Because the computational principle to predict clusters is that the similarity of intra-serotype strains should be separable from that of inter-serotype strains, now the mixed border will certainly lead to discrepancies between DENV genetic cluster (serotypes) and antigenic clusters.

As reference, the large-scale animal data from Leah's study (Katzelnick et al., 2015) were calculated similarly to show the difference between 4 intra- and 6 inter-groups (Figures 3A,B). The discriminable genetic border, but not the antigenic border, can be observed again in experiments as well. The agreeing results indicated that DENV can be clearly clustered into four groups genetically, but not antigenically. Thus, the traditional DENV antigenic cluster should be re-evaluated.

Then, all the pair-wised antigenic similarity of 1,143 historical strains were mapped into a clustering tree (Figure 3C), while different colors represent different DENV serotypes. It can be found that most of the intra-type strains tend to cluster together, which were consistent with the serotype classification, as in the case of serotype 1 and serotype 3. However, substantial number of strains are clearly clustered into other serotypes. For instance, a number of serotype 4 strains are grouped into serotype 2 and 3. More interesting, two different antigenic groups can be clearly demonstrated for DENV 2. Therefore, we would like to propose the further subtyping of DENV 2 into two sub-clusters, where DENV 2a was antigenically closer to serotype 4 rather than DENV 2b (Figure 3D).

Further, we clustered the antigenicity distance of DENV based on neutralization titer value from monkey experiments (Katzelnick et al., 2015). Because of the noise, the raw experimental data was cleaned as below: (1) null values were abandoned; (2) small and uncertain titers which labeled as “<10” were defined as 5; (3) the logarithm of each titer values was defined as the antigenic similarity of two compared strains. Antigenic clustering between remained pairs involving 28 DENV strains was illustrated in Supplementary Figure 4.

It can be found that besides four clusters representing four traditionally defined serotypes, a new cluster of DENV2 can be detected, which was antigenically closer to serotype 4 rather than serotype 2. This experiments result can support our proposal of two sub-clusters for DENV2.

## Discussion

The antigenic difference between DENV viruses plays essential role to the DENV epidemic control, vaccine-based prevention, and clinical treatment. In this paper, we built an accurate and efficient model to calculate the antigenic similarity for DENV strains based on mutated sequences of E proteins. To achieve that, we primarily considered the possible ADPs instead of all mutations in E antigens, not only for the reasons of computing efficiency, but also for predicting accuracy.

It is aware that not all mutations can cause antigenicity variation. After possible ADPs were derived where mutations could significantly affect the antigenicity, ADPs were further clustered into spatial patches for detecting potential epitope regions based on geometric distance on protein surface. It is noted that ADPs were calculated from experimental data previously accumulated. More abundant experimental data will lead to more accurate model. Despite the creditability of our model, the range of ADPs might be refreshed, and slightly adjusted with the future accumulation of latest binding assays, so as the minor changes of antigenic grouping.

Apart from the contribution of ADPs, the performance of our model is also contributed by full consideration of PSSM profile and the physic-chemical environment around the ADPs. Here, the PSSM generated by PSI-BLAST (Altschul et al., 1997) could provide a detailed description on evolution pressure of ADPs at sequence level. Moreover, the physic-chemical environment described by amino acid indexes are also considered to better reflect the micro-environment variations between two compared strains (Qiu et al., 2016). Thus, by incorporating PSSM profiles and PCDs, our model could better predict the antigenicity variation of DENV strains.

It was reported that, many DENV isolates are antigenic similar to those viruses from different types rather than those from the same type (Katzelnick et al., 2015). In this paper, we explained the reasons why canonical DENV types are not antigenically homogenous. Both data of experiments and historically published sequences showed that, the mutation accumulation is discrete but the antigenicity variation of mutants tends to be continuous among the DENV mutant populations (Supplementary Figure 5). The discrete genetic distance between intra- and inter-groups make it easy to define DENV subgroups but that may not correlate with the antigenic similarity. Thus, we suggest the re-consideration of the traditional serotype definition via DENV antigenic similarity instead of genetic distance. Our model provides convenient way to calculate the relative antigenicity difference.

In summary, we established as a fast and efficient model for DENV antigenicity based on sequence input of E antigens. With the improvement of ADPs updating and incorporation of additional antigens, it will be possible to establish an on-line tool to serve the purpose of epidemic monitoring and broad-spectrum vaccine design of DENV.

## Data Availability

Sequence data of dengue virus envelop protein (E) were derived from NCBI virus resources at https://www.ncbi.nlm.nih.gov/genomes/VirusVariation/Database/nph-select.cgi?cmd=database&taxid=12637.

## Author Contributions

JQ and TQ developed the algorithm. TQ and YS wrote the manuscript. JQ constructed the computational model. TQ and ZJ supervised the whole project and modified the manuscript. All authors read and approved the final manuscript.

### Conflict of Interest Statement

The authors declare that the research was conducted in the absence of any commercial or financial relationships that could be construed as a potential conflict of interest.
