# Supplementary material for: In-silico Antigenicity Determination and Clustering of Dengue Virus Serotypes
Source: Front Genet. 2018 Dec 7;9:621. doi: 10.3389/fgene.2018.00621 (PMC6292942; doi:10.3389/fgene.2018.00621)
Supplement: Supplementary file 1 [file Data_Sheet_1.docx]

Supplementary Table 1. Antigenicity-dominant positions for DENV and four serotypes.

| Serotype | Sites |
| --- | --- |
| DENV antigenic sites | 4，6，7，8，18，19，22，29，51，52，53，62，63，64，65，68，72，80，88，90，91，93，96，112，118，122，124，126，128，140，143，147，148，149，150，155，157，160，163，164，168，171，173，176，181，183，202，203，204，221，226，227，228，229，232，236，243，247，249，262，272，277，280，293，297，300，303，304，305，307，308，309，313，323，327，329，330，331，338，339，342，343，345，347，355，359，360，364，377，379，383，384，385，386，389，390，394 |
| DENV_1 antigenic sites | 15，18，50，51，88，126，132，140，145，148，150，169，171，180，200，226，233，305，320，338，339，342，343，345，346，347，357，359，360，385 |
| DENV_2 antigenic sites | 8，29，36，37，52，53，68，83，90，91，122，124，126，149，162，164，171，174，176，181，202，204，226，236，247，262，277，280，297，303，307，330，331，345，346，347，360，365，379，390 |
| DENV_3 antigenic sites | 4，7，8，22，29，50，52，62，63，65，90，118，120，122，126，140，155，164，167，170，180，186，202，228，233，251，265，297，304，329，330，331，338，345，351，353，355，358，359，364，365，382，383，384，385，386，394 |
| DENV_4 antigenic sites | 7，22，46，64，65，90，96，118，120，122，132，143，147，148，155，169，171，181，183，202，203，221，227，232，265，280，329，330，340，342，351，355，364，383，384，385，386 |

Supplementary Table 2. Evaluation of neighborhood region.

|  | Correlation coefficient | RMSE |
| --- | --- | --- |
| 1Å neighbor | 0.717 | 0.991 |
| 2Å neighbor | 0.711 | 1 |
| 3Å neighbor | 0.687 | 1.032 |
| 4Å neighbor | 0.588 | 1.151 |
| 5Å neighbor | 0.592 | 1.147 |


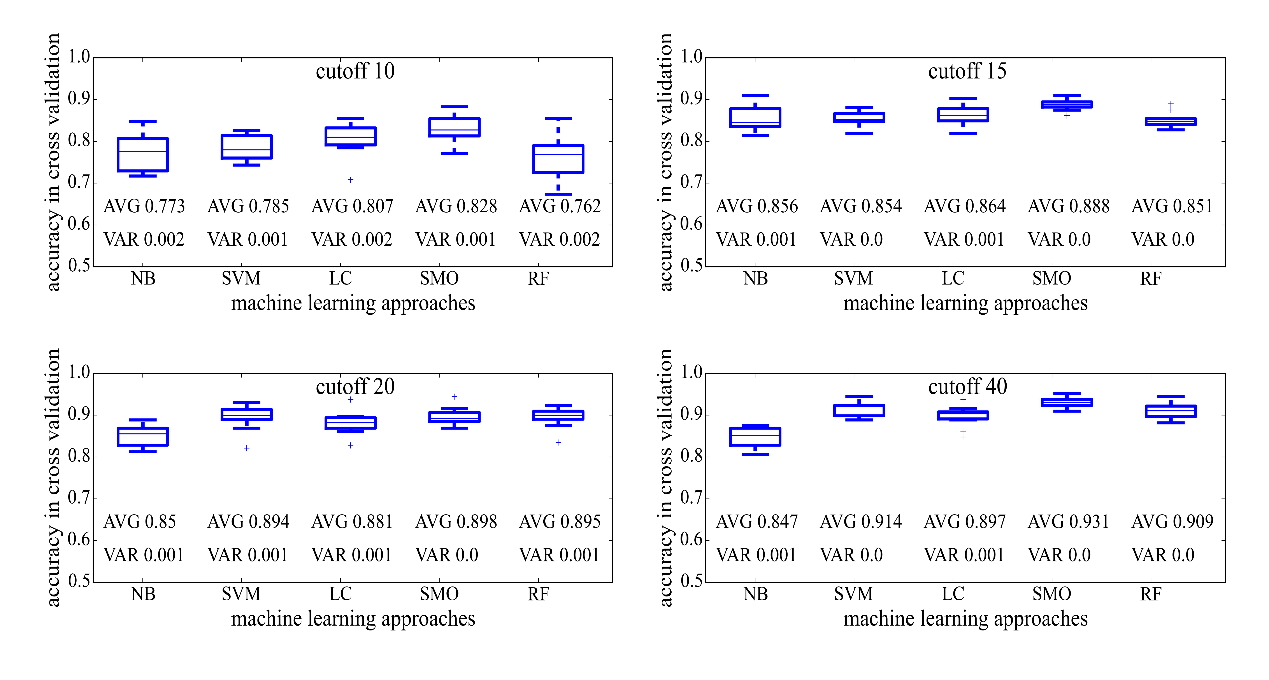


Supplementary Figure 1. Classification accuracy of different machine learning models under different cutoffs through 10-fold cross validation. Different titer cutoffs were set as 10, 15, 20 and 40 based on neutralization values from experimental work. For each machine learning approach, distribution of accuracy value on 10-fold cross-validation was shown on boxplot. AVG refers to the average of accuracy values and VAR refers to variance of accuracy value.


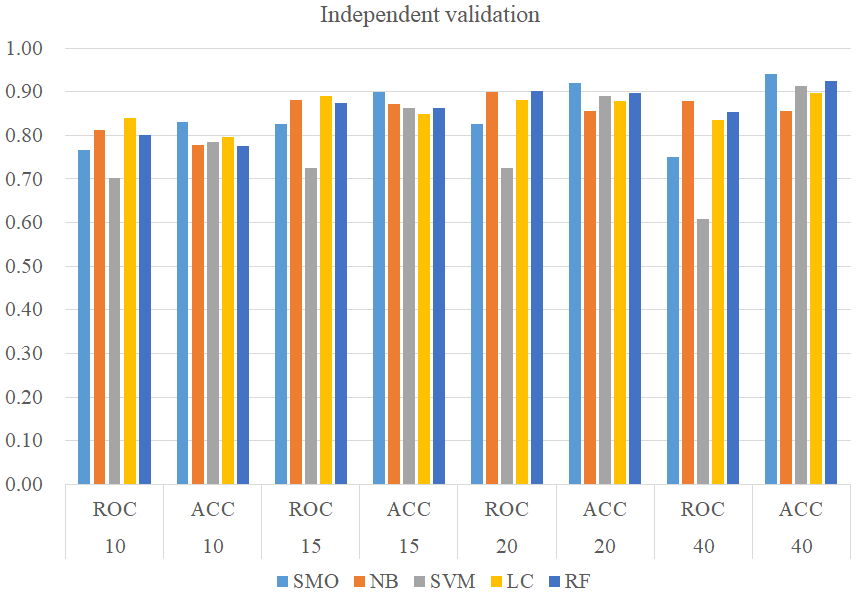


Supplementary Figure 2. Independent-validation performance of qualitative model constructed by Sequential Minimal Optimization, Naïve Bayes, Support Vector machine, Logistic, and Random Forest.


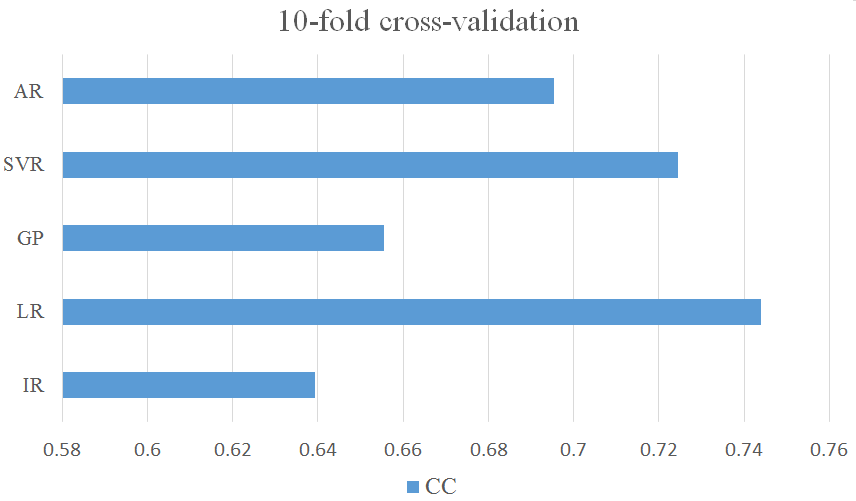


Supplementary Figure 3. Cross-validation performance of quantitative model constructed by Additive Regression (AR), Support Vector Regression (SVR), Gaussian Processes (GP), Linear Regression (LR) and Isotonic Regression (IR).


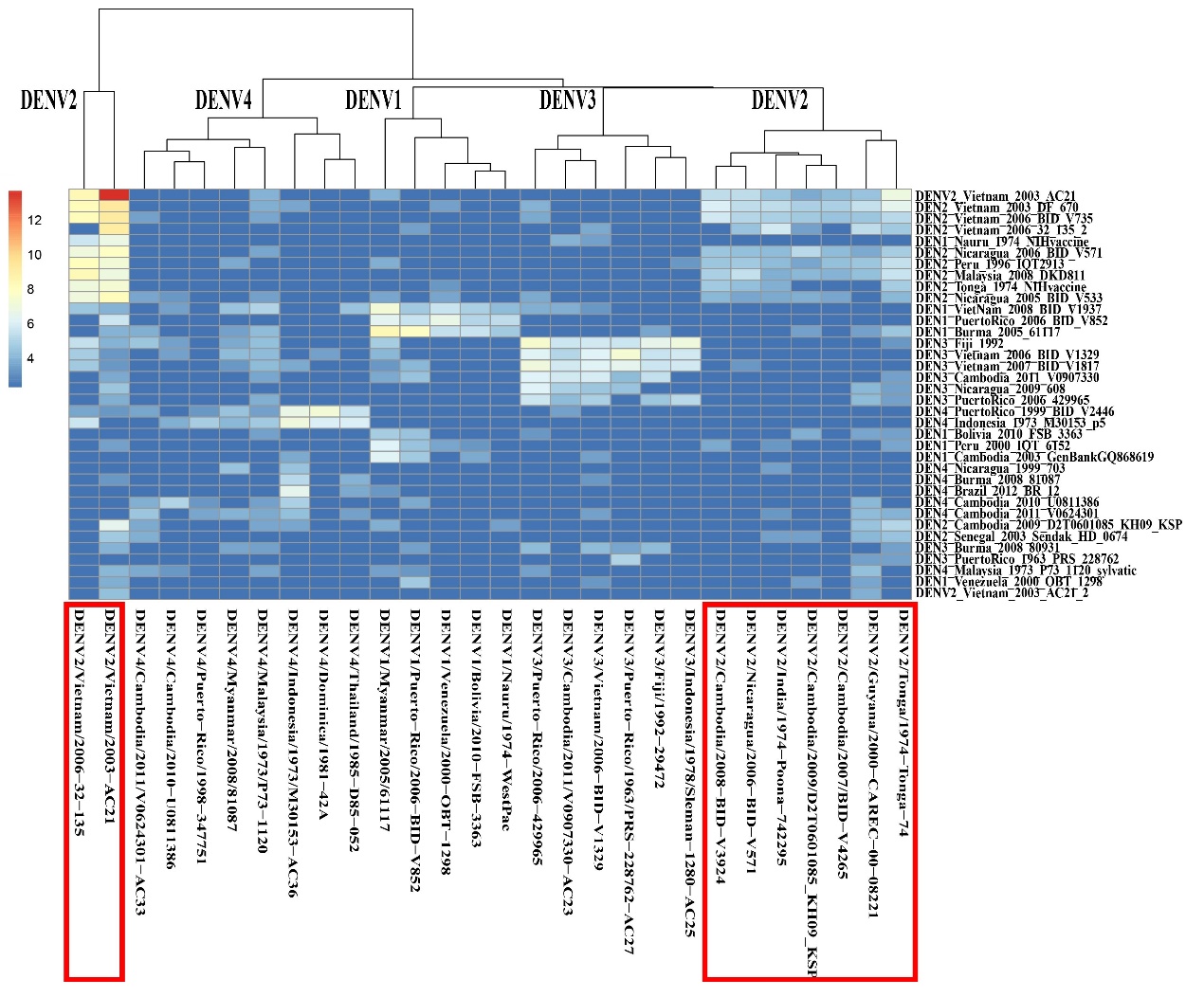


Supplementary Figure 4. Antigenic cluster of 28 DENV strains based on experimental results from Katzelnick’s work ([Katzelnick et al., 2015](#_ENREF_1)). The heatmap was made based on the logarithm of neutralization titer values. Higher values refer to higher antigenic similarity, while DENV strains were clustered based on antigenic similarity. Red boxes indicated the location of DENV2.


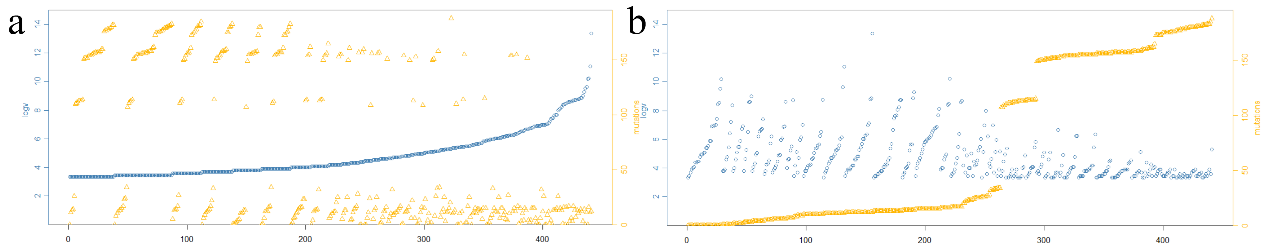


Supplementary Figure 5. Antigenicity and sequence analysis of DENV. (a) Relationship between antigenic similarity and genotype variations based on experimentally evaluated DENV strains according to ascending orders of antigenic similarity. (b) Relationship between antigenic similarity and genotype variations based on experimentally evaluated DENV strains according to ascending orders of amino acid mutations.

**REFERENCES**

Katzelnick LC, Fonville JM, Gromowski GD, Bustos Arriaga J, Green A, James SL, Lau L, Montoya M, Wang C, VanBlargan LA, Russell CA, Thu HM, Pierson TC, Buchy P, Aaskov JG, Munoz-Jordan JL, Vasilakis N, Gibbons RV, Tesh RB, Osterhaus AD, Fouchier RA, Durbin A, Simmons CP, Holmes EC, Harris E, Whitehead SS and Smith DJ (2015) Dengue viruses cluster antigenically but not as discrete serotypes. Science 349:1338-1343.
